# Long-term maintenance of diphtheria-specific antibodies after booster vaccination is hampered by latent infection with Cytomegalovirus

**DOI:** 10.1186/s12979-017-0099-y

**Published:** 2017-06-26

**Authors:** Birgit Weinberger, Michael Keller, Beatrix Grubeck-Loebenstein

**Affiliations:** Institute for Biomedical Aging Research, Universität Innsbruck, Rennweg 10, A-6020 Innsbruck, Austria

**Keywords:** Cytomegalovirus, Diphtheria, Antibody maintenance, Elderly

## Abstract

Many currently used vaccines are less immunogenic in the elderly compared to young adults. The impact of latent infection with Cytomegalovirus (CMV) on vaccine-induced antibody responses has been discussed controversially. We have demonstrated that recall responses to diphtheria vaccination are frequently insufficient in elderly persons and that antibody concentrations decline substantially within 5 years. In the current study we show that within a cohort of healthy elderly (*n* = 87; median age 71 years, range 66–92) antibody responses to a booster vaccination against diphtheria do not differ between CMV-negative and CMV-positive individuals 4 weeks after vaccination.. However, the goal of diphtheria-vaccination is long-term protection and this is achieved by circulating anti-toxin antibodies. Diphtheria-specific antibody concentrations decline faster in CMV-positive compared to CMV-negative older adults leading to an increased proportion of persons without protective antibody concentrations 5 years after booster vaccination and endangering long-term protection. This finding could be relevant for vaccination schedules.

**Electronic supplementary material:**

The online version of this article (doi:10.1186/s12979-017-0099-y) contains supplementary material, which is available to authorized users.

Aging is associated with characteristic changes of the immune system, collectively termed immunosenescence, which contribute to increased incidence and severity of infection [1] and to decreased immunogenicity and efficacy of vaccination [2, 3]. One hallmark of immunosenescence is the involution of the thymus, i.e. the gradual replacement of functional thymic tissue by fat [4], leading to a severely decreased output of newly generated naïve T cells and as a consequence to the loss of naïve T cells in lymphoid organs and in the periphery [5, 6]. Concomitantly, highly differentiated effector T cells accumulate resulting in alterations of the cytokine profile and in decreased diversity of the T cell repertoire [7]. Several studies have demonstrated that latent infection with the human β-herpesvirus Cytomegalovirus (CMV), which is prevalent without clinical symptoms in 60–100% of the adult population, aggravates age-related changes of the T cell compartment [6, 8, 9] and is part of the “immune risk phenotype”, which predicts 2-year mortality in the very elderly [10]. Epidemiological studies indicate that CMV-seropositivity is associated with a slight increase in overall mortality [11, 12]. It has been demonstrated recently that CMV also affects B cell function. CMV-seropositivity is associated with decreased switched memory B cells and in vitro activation of activation-induced cytidine deaminase (AID), which are predictors for successful influenza vaccination [13]. In addition, CMV drives the expansion of CD56^dim^CD57^+^NKG2C^+^ NK cells [14], skewing the NK cell repertoire to more cytotoxic responses at the expense of cytokine-driven functions. As a result, in vitro NK cell responses to influenza and pertussis vaccine antigens are impaired [15]. The impact of CMV-seropositivity or the level of CMV-specific antibodies on immune responses after vaccination is controversially discussed. Some studies report that antibody responses to vaccines, e.g. against influenza, are lower in CMV-seropositive older individuals, or in persons with high concentrations of CMV-specific antibodies [16, 17]. In contrast, other studies did not observe an impact of CMV-infection on vaccine-induced immune responses against influenza or *Streptococcus pneumoniae* [18, 19]. No data are available regarding the impact of latent infection with CMV on the long-term maintenance of vaccine-induced antibodies. We therefore addressed this question using data from one of our previously published studies on the maintenance of tetanus- and diphtheria-specific antibodies after vaccination of an elderly cohort [20, 21]. We have demonstrated that recall responses to diphtheria vaccination are frequently insufficient in elderly persons and that antibody concentrations decline substantially within 5 years. Two hundred two older adults (>60 years) received a single shot of tetanus and diphtheria containing vaccine and antibody concentrations were measured before and 4 weeks after vaccination [20]. Five years later 87 persons of the original cohort were willing to participate in a follow-up study and received a second dose of tetanus and diphtheria vaccine. Analysis of the long-term persistence of tetanus- and diphtheria-specific antibodies was performed for this sub-cohort [21]. We demonstrated that tetanus- and diphtheria-specific antibody concentrations had dropped to the level before the first vaccination within 5 years. As tetanus-specific antibody concentrations were generally higher, almost all participants were still protected. In contrast, 45% of our elderly cohort did not have protective levels of diphtheria-specific antibodies 5 years after re-vaccination (Table 1).

**Table 1:** Number and percentage of persons with antibody concentrations below the protective level

	1st vaccination		2nd vaccination	
	day 0	day 28	day 0	day 28
Tetanus	10 (12%)	0	9 (10%)	0
Diphtheria	55 (65%)	9 (11%)	39 (45%)	6 (5%)

Number and percentage of persons (>60 years) with antibody concentrations below the protective level (≤0.1 IU/ml) before and 4 weeks after vaccination against tetanus and diphtheria. Data are shown for two vaccinations given at a 5-year interval. Data from [21]

As protection against tetanus was generally high, we focused on diphtheria in the current study and aimed to evaluate the impact of latent infection with CMV on the long-term maintenance of diphtheria-specific antibodies. Antibody concentrations were similar for CMV-negative and CMV-positive participants before the first vaccination. The vaccination history was very variable at this time point probably masking a potential impact of latent CMV-infection. Antibody concentrations increased to the same extent in both groups after vaccination. Five years later antibody concentrations had dropped to the original levels in the CMV-positive group. In contrast, antibody concentrations were still higher than baseline for the CMV-negative group. The difference between CMV-negative and CMV-positive participants was statistically significant at this time point. After the second vaccination, antibody concentrations increased to similar levels in both groups (Fig. 1a).

**Fig. 1:**
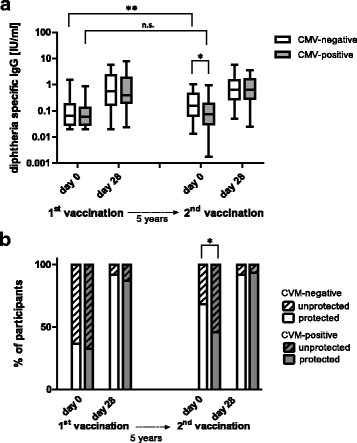
Antibody concentrations and percentage of persons with protective antibody concentrations. Diphtheria-specific antibody concentrations were measured by ELISA prior to and 4 weeks after vaccination. Two doses of diphtheria toxoid containing vaccines were applied in a 5-year interval. **a** Boxes show 25th and 75th percentiles, the median value is indicated. Whiskers depict 5th and 95th percentiles. Only donors, for whom data points were available at all 4 time points are included in the analysis. Differences between CMV-negative and CMV-positive groups were calculated using Mann-Whitney U test. Differences between day 0 (1st vaccination) and day 0 (2nd vaccination) were calculated using Wilcoxon signed-rank test. *p < 0.05; **p < 0.01; n.s.: not significant. **b** Percentage of participants with antibody concentrations above (protected, solid bar) or below (unprotected, dashed bar) levels considered to be protective (0.1 IU/ml). The CMV-negative group is shown in white, the CMV-positive group in gray. Differences in the percentage of persons protected / unprotected were calculated using Pearson Chi-square test. *p < 0.05; **p < 0.01; n.s.: not significant

These differences in antibody concentrations were also reflected in the percentage of persons with antibody concentrations below the level considered to be protective (≤0.1 IU/ml). Five years after the first vaccination, 54% of the CMV-positive donors were not protected, whereas only 33% of the CMV-negative donors had antibody concentrations below the protective level at this time point. There were no differences in the levels of protection between CMV-positive and CMV-negative participants at the other time points (Fig. 1b). The difference in antibody concentrations observed prior to the second vaccination is due to a more pronounced decline of antibody concentrations over 5 years in the CMV-positive cohort (Fig. 2).

**Fig. 2:**
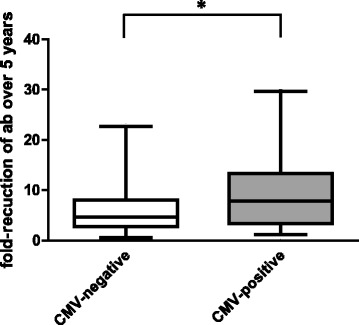
Decrease of diphtheria-specific antibody concentrations over 5 years. Depicted is the fold-reduction of diphtheria-specific antibodies from 4 weeks after the first vaccination until immediately before the second vaccination (5 years). Boxes show 25th and 75th percentiles, the median value is indicated. Whiskers depict 5th and 95th percentiles. The differences between CMV-negative and CMV-positive groups was calculated using Mann-Whitney U test. *p < 0.05

T and B cells subsets of 19 CMV-negative and 18 CMV-positive participants were analyzed by flow cytometry at the time of the second vaccination. CMV-related changes of T cell subsets, namely a decrease of naïve and an increase of effector T cell subsets in CMV-positive persons have been described [6, 8, 9] and could be confirmed in our cohort. In contrast, no differences of B cell subsets were observed (Table 2). Age-related changes in the B cell compartment include a loss of naïve B cells and an accumulation of highly differentiated double negative (IgD^−^CD27^−^) cells [22], which were also termed exhausted, and have been described to be associated with lower responses to influenza vaccination [23]. However, in accordance with our data, it has previously been reported that the influence of latent CMV infection on the composition of B cell subsets is only minimal [24], but that CMV infection might influence the B cell repertoire [25].

**Table 2:** T cell and B cell subsets in CMV-negative and CMV-positive individuals

		CVM-negative	CMV-positive	p
		mean ± SD	mean ± SD	
CD4^+^	naïve	63.0 ± 12.4	52.0 ± 15.5	0.029*
	central memory	36.2 ± 12.5	38.5 ± 15.3	0.605
	effector memory	0.6 ± 0.4	5.3 ± 4.2	<0.0001**
	TEMRA	0.7 ± 0.6	4.7 ± 5.2	<0.0001**
CD8^+^	naïve	42.2 ± 16.2	28.9 ± 15.4	0.024*
	central memory	25.4 ± 9.7	17.9 ± 14.0	0.005**
	effector memory	8.9 ± 8.8	8.4 ± 7.3	0.939
	TEMRA	24.5 ± 16.7	45.7 ± 17.5	0.001**
B cells	IgM^+^	19.5 ± 8.6	16.8 ± 5.6	0.286
	IgD^+^	67.7 ± 11.9	64.6 ± 12.5	0.564
	IgG^+^	11.2 ± 8.0	11.4 ± 4.4	0.323
	memory IgG	8.3 ± 6.1	8.4 ± 3.6	0.485
	exhausted	9.4 ± 2.6	11.2 ± 6.8	0.862

Percentage of naïve (CD45RO^−^CD28^+^), central memory (CD45RO^+^CD28^+^), effector memory: (CD45RO^+^CD28^−^), and TEMRA (CD45RO^−^CD28^−^) cells within CD3^+^CD4^+^ or CD3^+^CD8^+^ cells, respectively and of IgM^+^, IgD^+^, IgG^+^, memory IgG (CD27^+^IgG^+^, and exhausted (IgD^−^CD27^−^) B cells within CD20^+^ cells. Shown are mean values and standard deviation (SD) from 19 CMV-negative and 18 CMV-positive individuals. Differences between CMV-negative and CMV-positive groups were calculated by Mann-Whitney-U test. *p < 0.05; **p < 0.01

summary, our data show that diphtheria-specific antibody concentrations decline faster in CMV-positive compared to CMV-negative older adults leading to an increased proportion of persons without protective antibody concentrations 5 years after booster vaccination and endangering long-term protection. This finding could be relevant for vaccination schedules. One possible reason for the faster decline of antibody concentrations might be an impaired maintenance and/or survival of long-lived plasma cells in the bone marrow. We have previously reported a decrease of diphtheria-specific plasma cells in the bone marrow with age [26], but the CMV-status was not taken into consideration in this small cohort. Recent data in our laboratory showed an increase of inflammatory and oxidative stress parameters in the bone marrow of older patients and at the same time a decrease of IL-7 and a proliferation-inducing ligand (APRIL), which is a survival factor for plasma cells [27]. The impact of latent CMV-infection on the bone marrow microenvironment and the antigen-experienced lymphocytes residing there is not yet known.

## Materials and methods

### Study cohort

For this study the 87 persons, who completed the 5-year follow-up and received two vaccinations against tetanus and diphtheria were included. In accordance with the original study protocol persons with chronic viral infection (Human Immunodeficiency virus, Hepatitis B virus, Hepatitis C virus), transplant recipients and patients under immunosuppressive or chemotherapy were excluded. Routine laboratory parameters (liver and kidney function, blood count) were determined. All participants were shown to be in good health and there were no differences between CMV-negative and CMV-positive persons. Table 3 shows the patient characteristics for the CMV-negative and the CMV-positive sub-cohort.

**Table 3:** Patient characteristics

	CMV-negative	CMV-positive	p
n (%)	39 (44.8%)	48 (55.2%)	-
age (median, range)	71 (66–92)	71 (67–89)	0.777^a^
female (%)	24 (61.5%)	25(50.0%)	0.282^b^
BMI (median, range)	24.8 (19.5–37.3)	26.1 (16–34.2)	0.155^a^

^a^Mann-Whitney-U test or

^b^Pearson Chi-square test was used to determine differences between CMV-negative and CMV-positive groups

### Determination of IgG antibody concentrations

Microtiter plates were coated with 1 μg/ml diphtheria toxoid (Statens Serum Institute) and blocked with 0.01 M Glycin. Serum samples were tested in duplicates. Peroxidase-labeled rabbit anti-human IgG (Chemicon/Millipore) antibody was used as secondary antibody. IgG antibodies were quantified in IU/ml using standard human anti-diphtheria serum (NIBSC). The detection limit of the assays used was 0.01 IU/ml and values below the limit of detection were set to 0.005 IU/ml. Antibody concentrations above 0.1 IU/ml were considered as protective.

Antibodies against Cytomegalovirus (CMV) were determined using a commercially available ELISA Kit (Siemens). Reciprocal titers above 231 were considered positive.

### Flow cytometry

PBMC were washed with PBS and stained with anti-CD3-PE-Cy7 (Biolegend), anti-CD4-PerCP (BD Pharmingen), anti-CD8-PE (BD Pharmingen), anti CD28-APC (Biolegend), anti CD45RO-FITC (BD Pharmingen), anti-CD20-PerCP (Biolegend), anti-CD27-APC-Cy7(Biolegend) and anti-IgD-FITC (BD Pharmingen) antibodies for 20 min, 4 °C in the dark. After washing with PBS, cells were analyzed using a FACS Canto II cytometer and FACSDiva software (BD).

### Statistical analysis

Comparisons between two independent groups (CMV-negative vs. CMV-positive) were calculated using Mann-Whitney U test. Differences between paired samples (different time points) were calculated using Wilcoxon signed-rank test. The distribution of categorical data (e.g. protected/unprotected) was calculated using the Pearson Chi-square test. *p* < 0.05 was considered significant for all tests.

## Additional file

Additional file 1: Data table. (XLSX 23 kb)
